# Characterization of the Noise Induced by Stimulated Brillouin Scattering in Distributed Sensing

**DOI:** 10.3390/s20154311

**Published:** 2020-08-02

**Authors:** Jaffar Emad Kadum, Cheng Feng, Thomas Schneider

**Affiliations:** THz Photonics Group, Institut für Hochfrequenztechnik, Technische Universität Braunschweig Schleinitzstr. 22, 38106 Braunschweig, Germany; cheng.feng@ihf.tu-bs.de (C.F.); thomas.schneider@ihf.tu-bs.de (T.S.)

**Keywords:** Brillouin optical time-domain analyzer, noise analysis, Brillouin scattering, spontaneous-signal beating noise

## Abstract

The excess noise due to stimulated Brillouin scattering (SBS) in gain and loss-based Brillouin optical time-domain analyzers (BOTDA) has been investigated theoretically and experimentally for the first time to the best of our knowledge. This investigation provides a full insight to the SBS-induced noise distribution, which mainly comes from phase-to-intensity conversion noise and the beating noise between the probe wave and spontaneous Brillouin scattering. A complete theoretical model, which is in good agreement with the experimental results, is presented to describe the noise. The results show that a loss-based BOTDA setup gives a better noise performance than a gain-based one in both time and frequency domain. The SBS-induced noise has been characterized in dependence on the pump and probe power and the spatial resolution.

## 1. Introduction

Due to its ability for long range distributed sensing of temperature and strain with high spatial resolution, Brillouin optical-time domain analysis (BOTDA), has been recognized as one of the most attractive research topics in the field of distributed optical sensing [1]. The BOTDA employs stimulated Brillouin scattering (SBS), i.e., a nonlinear effect in standard optical fibers, where a pulsed pump optical wave counter-propagates to a probe continuous wave (CW) [2]. The interaction between the pump and probe wave excites an acoustic wave through electrostriction, which acts as a moving density wave [3]. This acoustic wave is responsible for the power coupling between the two counter-propagating optical waves. Brillouin scattering occurs when the pump wave is backward scattered on this acoustic wave, giving rise to frequency down-shifted Stokes and frequency up-shifted anti-Stokes components. The frequency shift of scattered waves, which is defined as Brillouin frequency shift (BFS), depends on the temperature and strain of the fiber [2]. Thus, this BFS, which is around 11 GHz in standard single mode fibers (SMF) in the C-band of optical telecommunications [4], is the parameter which will be measured in such a sensor. The pulsed pump wave generates a gain for the frequency down-shifted probe wave. Consequently, optical power is transferred from the pump to the probe. If another CW is propagating in the fiber with a frequency upshift in the same range, this upshifted wave will transfer optical power to the pump. Thus, the pulsed pump wave generates a loss for the upshifted counter-propagating wave [5]. The frequency offset between pump and probe waves determines the power transfer efficiency between them. The highest optical power transfer takes place when the frequency offset equals to the BFS of the fiber. Thus, the Brillouin gain spectrum (BGS) or Brillouin loss spectrum (BLS) can be constructed by scanning the pump–probe frequency offset.

Due to its ability to measure temperature and strain with standard telecom fibers, BOTDA is a promising technique in industrial applications such as the health monitoring of large structures, including oil and gas pipeline leakage detection and monitoring of long railway and high voltage transmission lines [6,7]. Especially for these scenarios, the BOTDA sensor has to work over long distances, which requires a very high signal-to-noise ratio (SNR) for the detected probe wave. Furthermore, the measurement accuracy, which is defined as the accuracy of the BFS estimation from the reconstructed Brillouin gain or loss spectrum, also depends on the SNR of the retrieved probe wave [8]. A high spatial resolution of the sensor requires short pump pulses, but these short pump pulses result in a very short interaction length between pump and probe. Thus, there is a trade-off between spatial resolution and gain and therefore the SNR and sensing range [9]. To increase the SNR of the retrieved probe, either the detected signal is amplified, or the noise level is reduced. To amplify the detected signal, the pump and probe power shall be increased. Nevertheless, the pump power is limited by the fiber nonlinearities such as modulation instability [10] and the probe power is limited by pump depletion, which leads to nonlocal effects [11]. Many methods, such as Raman amplification [12,13], pump pulse coding techniques [14] and self-heterodyne detection [15], have been proposed to enhance the signal level in BOTDA. However, these techniques either complicate the setup or increase the measurement time. Since the system noise degrades the SNR as well as increases the BFS estimation uncertainty [8], the detected BOTDA probe is usually averaged thousands of times to reduce the noise. The averaging increases the measurement time significantly and limits real-time acquisition, particularly in dynamic sensing applications [16,17]. Many approaches like wavelet de-noising techniques [18], 2D and 3D image restoration [19] and the employing of radio frequency (RF) low pass filtering [20] have been applied to reduce the noise level in BOTDA. Nevertheless, these methods might increase the post-processing time.

Noise in BOTDA can be classified into classical noise [21,22,23], which results from the laser and detector, and noise originated by the pump–probe interaction [24] which can be defined as SBS-induced noise (SBiN). Classical noise sources such as shot noise, thermal noise and laser relative intensity noise (RIN) are flat and proportional to the sensor bandwidth and can be alleviated by optimizing the detection bandwidth of the sensor [25]. The SBiN depends on the pump–probe frequency detuning and constitutes mainly due to the laser phase noise, which imposes phase-to-intensity (P-I) conversion noise [26,27] and the beating between the probe wave and waves generated by spontaneous Brillouin scattering (SpBS) [28,29,30].

Although both P-I and probe-SpBS beating can add considerable noise in specific applications [26,31], the Brillouin induced noise has not been well addressed in the literature of BOTDA sensors. In this paper, we present a theoretical model to describe the noise initiated during the pump–probe interaction and simulation results for loss-based BOTDA (L-BOTDA) and gain-based BOTDA (G-BOTDA). An experimental investigation has been carried out to validate the analytical model. The noise level in the Brillouin gain/loss spectrum for both G-BOTDA and L-BOTDA has been experimentally measured along the fiber. The study of SBS-induced noise provides a full insight to the noise behavior during frequency scanning, which might be important for the enhancement of the SNR in BOTDA sensors.

## 2. Noise Sources in BOTDA

**Figure 1:**
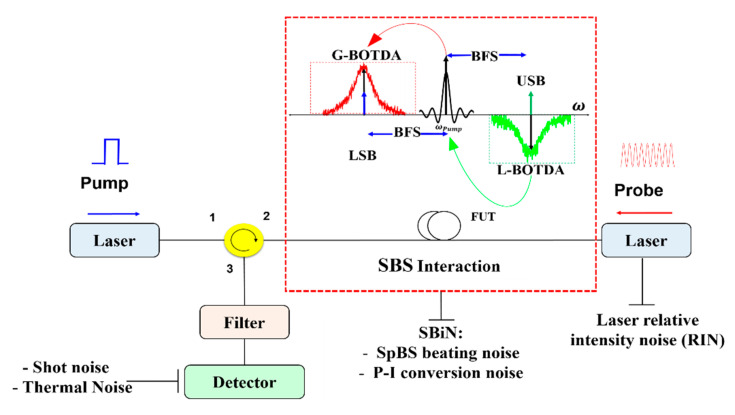
Schematic diagram of the BOTDA system with main noise sources. The inset depicts the SBS interaction between the pump and the probe waves in the frequency domain. FUT: fiber under test, LSB: probe lower frequency sideband, USB: probe upper frequency sideband, BFS: Brillouin frequency shift. The small blue and green arrows within the G-BOTDA and L-BOTDA band describe the spontaneous Stokes and anti-Stokes Brillouin component, respectively.

The noise sources for a typical BOTDA setup with a lower and upper frequency sideband probe wave can be seen in Figure 1. The major sources for the classical noise are from the detector and the laser. The sources in the detector include shot noise with the noise variance $\sigma_{sh}^{2}$, which originates due to the randomly generated photocarriers in the detector that leads to fluctuations in the photocurrent and thermal noise $\sigma_{th}^{2}$, which is a result of random thermal electron motions. The noise contribution of the laser is mainly relative intensity noise $\sigma_{RIN}^{2}$. Thus, the variance of the classical noise can be written as:

(1) $$\sigma_{CN}^{2}=\sigma_{sh}^{2}+\sigma_{th}^{2}+\sigma_{RIN}^{2}$$

The Brillouin scattering process in the fiber adds additional noise $\sigma_{SBiN}^{2}$ to the detected signal. This noise mainly consists of two contributions, i.e., the beating noise between the probe and the spontaneously scattered component $\sigma_{SpBS}^{2}$ and the phase-to-intensity conversion noise $\sigma_{p-i}^{2}$. Thus, the total noise in the fiber during the SBS process is the summation of the classical noise and the noise originated by the scattering process:

(2) $$\sigma_{T}^{2}=\sigma_{CN}^{2}+\sigma_{SBiN}^{2}$$

Other noise sources, such as the intensity noise transferred from the pump wave to the probe and Rayleigh scattering, may be originated in the probe due to the pump propagation in the fiber. However, these noises can be neglected due to the low pump–probe intensity noise transfer [21]. The same holds for noise, generated by the anti-Stokes process, which might be transmitted from the upper frequency sideband to the probe and from there to the lower frequency sideband. The Rayleigh scattering has a flat spectrum with no impact on the measurement and can be eliminated by a simple filtering [21].

## 3. Theoretical Model

### 3.1. Probe-SpBS Beating Noise $\sigma_{SpBS}^{2}$

For very low input pump powers, the SBS is initiated by thermally initiated acoustic waves, leading to a frequency down-shifted Stokes and a frequency upshifted anti-Stokes spontaneous scattering [32]. Owing to the origin of this spontaneous scattering by thermal density fluctuations of the medium, it shows stochastic fluctuations [33,34].

In BOTDA, the Brillouin gain/loss spectrum is usually constructed by scanning the probe wave around the center of the BFS of the fiber. To compensate the pump depletion, usually a second probe wave counter-propagates with the pump wave [35]. In this case, the probe is a double sideband wave with a frequency separation of twice the BFS in the fiber (see the frequency diagram in Figure 1). Thus, the upper and lower frequency sidebands interact with the pump via Brillouin loss and gain, respectively. Then, the total optical field at port 3 of the circulator (yellow circle in Figure 1) is given by:

(3) $$\begin{array}{ll} E_{t}(t)= & E_{s}^{+}e^{i\left\{\left[\omega_{p}-\left(\omega_{BFS}\pm \Delta \omega \right)\right]t+\phi_{B}^{+}(t)\right\}}+E_{spBS}^{s}e^{i\left[\left(\omega_{p}-\omega_{BFS})t+\phi_{SpBS}^{+}(t\right)\right]}+\\ & E_{s}^{-}e^{i\left\{\left[\omega_{p}+\left(\omega_{BFS}\pm \Delta \omega \right)\right]t+\phi_{B}^{-}(t)\right\}}+E_{spBS}^{As}e^{i\left[\left(\omega_{p}+\omega_{BFS})t+\phi_{SpBS}^{-}(t\right)\right]} \end{array}$$

where $E_{s}^{+}$ and $E_{s}^{-}$ are the electrical fields of the amplified and attenuated probe sidebands, respectively, whereas $E_{spBS}^{s}$ and $E_{spBS}^{As}$ are the fields of the spontaneous Stokes and anti-Stokes components. $\phi_{B}^{+}$ represents the phase of the probe lower frequency sideband and $\phi_{B}^{-}$ is the phase of the upper frequency sideband. *ω_p_*/2*π*, *ω_BFS_*/2*π* and Δ*ω*/2*π* represent the pump frequency, BFS of the fiber and frequency detuning around the BFS, respectively. $\phi_{SpBS}^{\pm }$ are the random phases of the spontaneous Stokes and anti-Stokes Brillouin components. The complex Brillouin gain/loss coefficient is given by [36]:

(4) $$G_{B}^{\pm }(\omega )=\frac{\pm \frac{1}{2}g_{0}}{1-2i(\omega -\omega_{BFS})/\Gamma_{B}}$$

where $g_{0}$ is related to the inherent material SBS gain coefficient $g_{B}$ according to: g0=gBAeff where *A_eff_* represents the effective area of the optical fiber. The plus sign describes the gain spectrum, while the minus corresponds to the loss and Γ*_B_*/2*π* is their linewidth. For a CW pump, the linewidth is usually in the range of 20–30 MHz in SMF at 1550 nm and can be reduced with several methods [37,38,39,40]. Since BOTDA are working with pulses, which define the spatial resolution, the SBS linewidth is broader for a higher spatial resolution and vice versa. The imaginary part of Equation (4) is the Brillouin phase spectrum, which modifies the phase of the probe wave, while the real part represents the amplitude gain or loss spectrum that modifies the probe amplitude and follows a Lorentzian distribution.

In gain-based BOTDA, an appropriate fiber Bragg grating (FBG) filter is used to detect just the lower frequency sideband of the probe (first term in Equation (3)). However, the spontaneous Stokes component (second term in Equation (3)), is in the same band as the detected probe lower sideband. While in loss-based BOTDA, the spontaneous anti-Stokes component (last term in Equation (3)), is in the same band as the detected probe upper frequency sideband. Thus, in loss/gain based BOTDA, the probe upper/lower frequency sideband and the spontaneous anti-Stokes component reach the detector leading to a beating noise in the detected probe wave. Due to the weak birefringence of SMF, the state of polarization (SOP) of the spontaneous wave varies randomly along the fiber. To achieve a general analytical solution for the variance of the SpBS-probe beating noise, it is assumed that the SOP of the spontaneous and the probe waves is changing randomly along the fiber. Assuming that the spontaneous Brillouin component and the probe wave are uncorrelated, the noise variance imposed to the detected current in the photo diode (PD) by the Stokes and anti-Stokes spontaneous component is given by (for a detailed derivation, see the Appendix A):

(5) $$\sigma^{2}{}_{SpBS}={ℜ}^{2}P_{G/L}e^{-\alpha L}P_{SpBS}$$

where ℜ is the detector responsivity, *P_G/L_* is the probe power in gain/loss-based BOTDA, α is the loss coefficient, *L* is the total fiber length and *P_SpBS_* is the power of the spontaneous components, which can be calculated from [41]:

(6) $$P_{SpBs}=\frac{1}{2}S\gamma_{SBS}P_{p}e^{-\alpha z}\frac{1}{2}T\nu_{c}$$

where *S* is the fiber capture fraction, $\gamma_{SBS}$ is the Brillouin scattering coefficient, *P_p_* is the peak pump power, *T* the pump pulse width and $\nu_{c}$ is the speed of light in the fiber.

### 3.2. Phase-to-Intensity Conversion Noise $\sigma_{p-i}^{2}$

Due to the spontaneous emission in the laser, its output shows instantaneous phase fluctuations, which can be seen as noise [42]. The laser phase noise follows a Wiener–Levy nonstationary zero-mean Gaussian random statistical model [43]. In essence, the instantaneous frequency deviation is the time derivative of a time varying phase change and it is given by:

(7) $$f_{i}(t)=\frac{1}{2\pi }\frac{d\phi (t)}{dt}$$

To overcome the limitation imposed by the laser phase noise for two sources, all optical waves in a BOTDA system are usually derived from the same laser source. Nevertheless, at each fiber segment the phase difference between the counter-propagating pump and probe waves is changing during their Brillouin interaction. This phase fluctuation can be interpreted as an extra random frequency deviation Δ*ω_i_* between the pump and probe at a certain fiber position. Therefore, the Brillouin gain/loss experienced by the probe is also varying randomly (see Equation (4)) and the phase noise is translated into an intensity noise by the Brillouin interaction. The variance of this phase-to-intensity noise is [24]:

(8) $$\sigma_{p-i}^{2}=2{ℜ}^{2}\cdot {\left[\Delta \omega_{L}P_{G/L}e^{-\alpha L}p_{p}e^{-\alpha z}\Delta z\frac{\partial G_{B}(\Delta \omega )}{\partial \Delta \omega }\right]}^{2}$$

where $\Delta \omega_{L}$ is the laser linewidth and $\Delta z=\nu_{c}T/2$ is the spatial resolution. According to Equation (8), the P-I noise depends on the derivative of the Brillouin spectrum. Thus, it has to be zero at the center of the Brillouin frequency spectrum. Since the cumulated phase shift between pump and probe fields is a Gaussian variable and it is independent of fiber position, the P-I noise should be independent on the fiber length [27]. However, according to Equation (8), the P-I noise is proportional to the probe and pump power. Since probe and pump are attenuated along the fiber, the P-I noise should decrease with fiber length. For the SpBS-probe beating noise, the noise is proportional to the probe power, which is reduced for longer fibers as well.

### 3.3. Simulation Model

To investigate the SBS-induced noise using the theoretical analysis presented in Section 3.1 and Section 3.2, a simulation model is developed. As already discussed, in gain-based BOTDA the spontaneous Stokes Brillouin component is within the bandwidth of the probe (first and second term of Equation (3)) and both are detected by the PD. This leads to a beating between the probe and the spontaneous Stokes component in the detector. Based on Equations (5) and (8), the SBS-induced noise in gain-based BOTDA at each frequency detuning step υ is given by:

(9) $$\sigma_{G-BOTDA}^{2}(\nu )={ℜ}^{2}P_{G}e^{-\alpha L}P_{SpBs}+2{ℜ}^{2}\cdot {\left[\Delta \omega_{L}P_{G}e^{-\alpha L}p_{p}e^{-\alpha z}\Delta z\frac{\partial G_{B}^{+}(\Delta \omega )}{\partial \Delta \omega }\right]}^{2}$$

In loss-based BOTDA, the SBS contribution to the noise consists of P-I noise and the noise due to the beating between spontaneous anti-Stokes noise and the probe wave. Similar to Equation (9), the SBS-induced noise in loss-based BOTDA can be given by:

(10) $$\sigma_{L-BOTDA}^{2}(\nu )={ℜ}^{2}P_{L}e^{-\alpha L}P_{SpBs}+2{ℜ}^{2}\cdot {\left[\Delta \omega_{L}P_{L}e^{-\alpha L}p_{p}e^{-\alpha z}\Delta z\frac{\partial G_{B}^{-}(\Delta \omega )}{\partial \Delta \omega }\right]}^{2}$$

The simulation is carried out with the following steps. First, BOTDA traces at each frequency detuning step are simulated. Since the pump and probe power in the simulation as well as in the experiment are below the threshold of the modulation instability and pump depletion, the pump power is attenuated only due to the fiber losses. Therefore, the BOTDA trace at each frequency detuning step can be simulated by calculating the local Brillouin gain/loss at each fiber position *z* from [44]:

(11) $$G_{}^{\pm }(\omega ,z)=\exp\left[2p_{p}G_{B}^{\pm }(\omega ,z)\Delta z_{eff}\right]\cdot \exp(-\alpha z)$$

where $\Delta z_{eff}=\left[1-\exp(-\alpha \Delta z)\right]/\alpha$ represents the effective interaction length. In the second simulation step, the classical and SBS-induced noises are modeled. The SBS-induced noise is simulated by calculating the theoretical noise profiles from Equations (10) and (11), while the classical noise is assumed as additive white Gaussian noise. The used parameters of the simulation correspond to the experimental setup. Thus, the fiber length is 10.6 km, while the pump peak and probe power are 20 dBm and −14 dBm, respectively. Additionally, α = 0.046 km^−1^ (0.2 dB/km), *S* = 2.11 ×$\;10^{-3}$, $\gamma_{SBS}$ = 1.17 ×$\;10^{-6}$ m^−1^. The Brillouin spectrum linewidth Γ*_B_* is 40 MHz, $g_{0}$ is 0.2 (m·W)^−1^ and the laser linewidth is 2 MHz.

**Figure 2:**
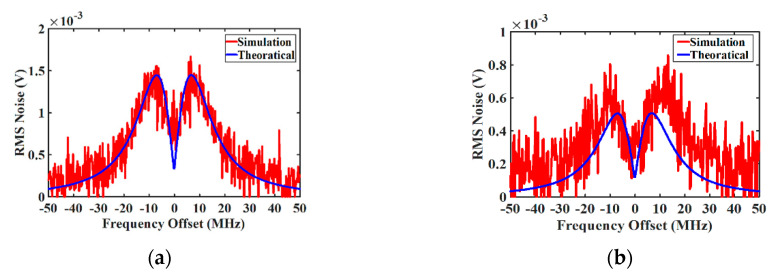
(**a**) Theoretical and simulated stimulated Brillouin scattering (SBS)-induced noise in gain-based Brillouin optical time-domain analyzers (G-BOTDA) and (**b**) loss-based Brillouin optical time-domain analyzers (L-BOTDA). In gain/loss-based BOTDA, the total theoretical noise (blue solid curve) consists of phase-to-intensity (P-I) noise and spontaneous Brillouin scattering (SpBS)-probe beating noise.

In BOTDA, usually short pump pulses are employed to get high spatial resolution, however, shorter pump pulses result in a lower SBS interaction length and therefore a lower noise contribution by SBS. Since here the SBS noise is investigated, the pump pulse width is chosen to be 175 ns. The theoretical and simulated SBS-induced noise for a gain-based BOTDA can be seen in Figure 2a and for the loss case in Figure 2b. As depicted in Figure 2a, for the gain case the dominant term of the SBS induced noise is the P-I noise. However, due to the contribution of spontaneous SBS emission, the noise is not zero in the center of the Brillouin gain spectrum. In gain-BOTDA, the probe power is amplified by the SBS gain and the power is transferred from the pump to the probe, leading to an increased beating noise. The noise transfer from the anti-Stokes component to the pump and from there to the probe is very low [21]. In loss-based BOTDA, however, the probe power is attenuated by the SBS loss and its power is transferred to the pump, therefore the beating noise, which is proportional to the probe power, is further reduced. Since the probe power is lower, even the P-I noise is reduced.

## 4. Experiment

**Figure 3:**
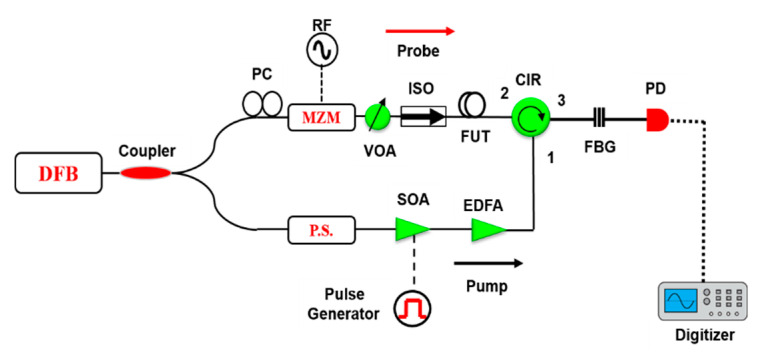
Experimental setup: RFG: radio frequency generator, PC: polarization controller, MZM: Mach–Zehnder modulator, VOA: variable optical attenuator, ISO: isolator, FUT: fiber under test, CIR: circulator, FBG: fiber Bragg grating, PS: polarization scrambler, SOA: semiconductor optical amplifier, EDFA: Erbium-doped fiber amplifier, PD: photodiode.

To verify the simulation results, an experimental noise measurement has been carried out using the setup in Figure 3. The output from a distributed feedback (DFB) laser at 1550 nm is split into two branches via a 90/10 optical coupler. In the upper branch, a double-sideband probe wave was formed by modulating the laser via a Mach–Zehnder modulator (MZM) driven by an RF generator, which scans the probe frequency in a range of 140 MHz in 0.25 MHz steps to reconstruct the Brillouin gain/loss spectrum. The bias point was set to obtain a probe signal with a double sideband and suppressed carrier. A polarization controller (PC) is inserted before the MZM to adjust the polarization of the probe wave and obtain maximum modulation efficiency. In order to obtain clean optical pulses, they are generated by a semiconductor optical amplifier (SOA) driven by an electrical pulse generator in the lower branch of the setup. The optical pulses are amplified by an erbium-doped fiber amplifier (EDFA). To compensate the polarization fading, a polarization scrambler was used before directing the pump pulses into the fiber, via an optical circulator. After the SBS interaction between pump and probe waves, the output signal was filtered by a narrowband FBG. For gain-based BOTDA, the lower frequency sideband of the probe is detected, while for a loss-based BOTDA measurement, the upper frequency sideband is selected. The RF probe signal was detected by a PD. After that, the probe signal was received by a digitizer with a sampling rate of 1 GSa/s and processed by a computer for averaging and further analysis. The experimental parameters are summarized in Table 1.

## 5. Results and Discussion

### 5.1. SBS-Induced Noise Calculation Method

**Figure 4:**
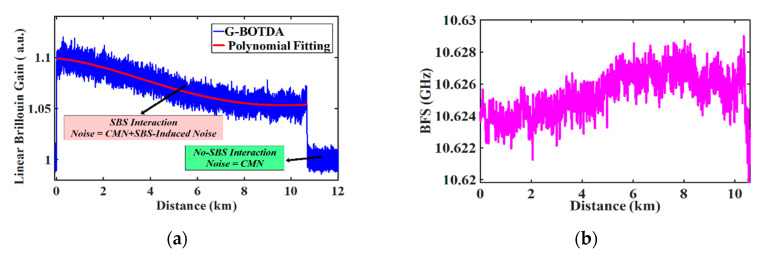
(**a**) Measured G-BOTDA time domain trace for 10.6 km fiber; (**b**) measured Brillouin frequency shift (BFS) profile of the fiber.

A measured gain-based BOTDA trace for a 10.6 km long fiber used in the experiment can be seen in Figure 4a. In a well-designed BOTDA, a high extinction ratio (ER) pump pulse wave is employed. Therefore, the SBS-interaction between the probe and pump pedestal can be neglected [45,46]. As a result, the noise outside the time domain trace consists of classical noise $\sigma_{CN}^{2}$ only. Consequently, the contribution of the SBS-induced noise $\sigma_{SBiN}^{2}$ can be quantized directly by subtracting the measured classical noise from the total measured noise $\sigma_{T}^{2}$ inside the Brillouin interaction region (see Figure 4a and Equation (2)). Due to the fiber losses, the pump power is reduced exponentially along the fiber and therefore the time domain traces should follow an ideal exponential curve. However, due to different strain regions along the fiber spool, there is a deviation from the exponential fitting. This effect is inevitable especially in long fibers, which always have a non-uniform BFS distribution. Figure 4b shows the measured BFS profile along the fiber, which clearly shows non-uniform strain regions. This extra strain causes an offset from the exponential function used to fit the time domain traces. In order to obtain an accurate noise calculation, a polynomial fitting (red curve in Figure 4a) is proposed to fit the time domain traces with arbitrary shape. Therefore, the noise of each time domain trace is calculated by the root mean square (rms) value at each frequency detuning step *v* according to:

(12) $$\sigma (\upsilon )=\sqrt[{}]{\frac{1}{n}\overset{n}{\underset{i=1}{\sum }}{\left(\left|\beta_{m}^{}(\upsilon )-\beta_{f}^{}(\upsilon )\right|\right)}^{2}}$$

where $\beta_{m}^{}$ is the measured value, $\beta_{f}^{}$ is the polynomial fitted value and *n* is the number of points given by the sampling rate.

### 5.2. Results

**Figure 5:**
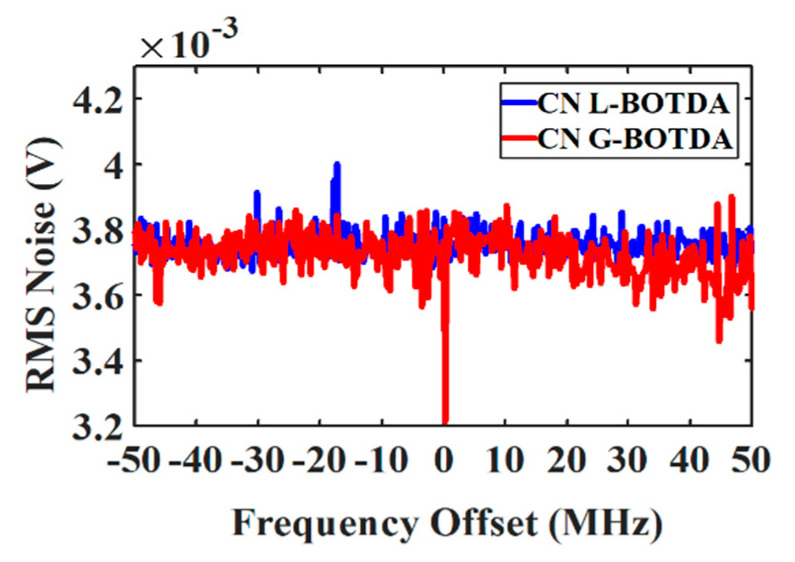
Experimental measurement of the classical noise (CN) with frequency detuning for G-BOTDA (red) and L-BOTDA (blue).

First, the classical noise (the noise outside the Brillouin interaction region) in dependence on the frequency detuning has been measured. As shown in Figure 5, the classical noise is almost constant over the whole Brillouin spectrum and it is almost equal for the gain and loss case. Moreover, due to the small Brillouin gain in BOTDA, power-dependent noises such as shot and RIN are determined by the probe wave and they are independent of the pump power and the spatial resolution [47].

**Figure 6:**
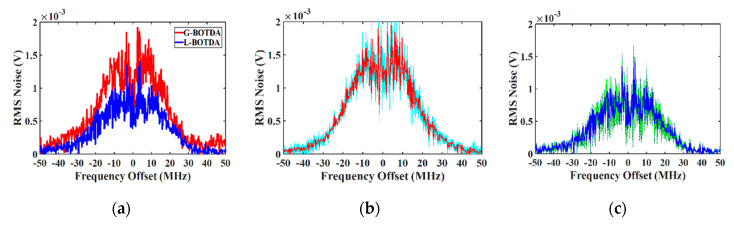
(**a**) Experimental measurement of the SBS-induced noise in dependence on frequency detuning for G-BOTDA and L-BOTDA; (**b**) the standard deviation error (cyan region) of the noise measurement (red curve) in G-BOTDA; (**c**) for L-BOTDA (green and blue).

To prove the concept of the noise measurement, four consecutive noise measurements have been carried out for both gain and loss-BOTDA. The rms measurements of the SBS-induced noise for the gain and loss cases are shown in Figure 6a. As can be observed, the SBS induced noise is lower than the classical noise by about 30% and 40% for gain and loss-based BOTDA, respectively, and the measurement follows the theoretical predictions. For the gain case, the noise consists of the phase to intensity and the spontaneous scattering beating noise. The first goes to zero in the center of the Brillouin gain, whereas the latter has its maximum in the center. Thus, the overall noise shows a dip at the center of the Brillouin spectrum. Since in the loss-based BOTDA case, the probe power is attenuated, both the P-I noise and the beating noise are reduced, leading to a lower SBS-induced noise than in gain-based BOTDA. The standard deviation error σ of the measurements has been calculated for each setup. Figure 6b,c illustrate the error bar as a shadow region (±σ) around the measured value.

### 5.3. Noise Dependence on Pump and Probe Power

**Figure 7:**
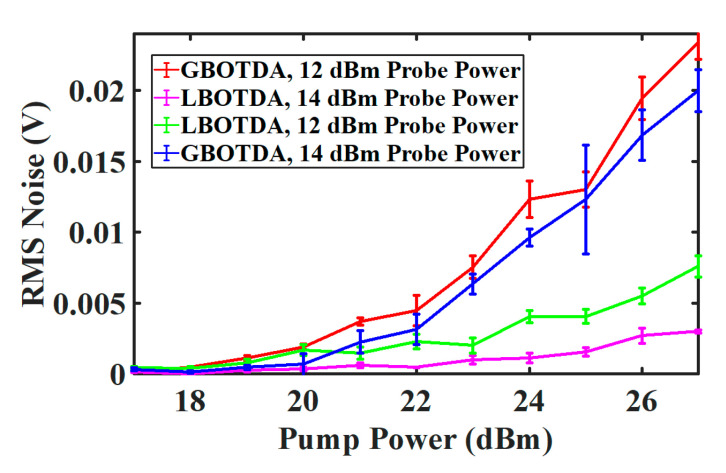
Experimental SBS-induced noise measurement in dependence on the pump power for G-BOTDA and L-BOTDA for −12 dBm and −14 dBm probe power. The error bars represent the standard deviation error of four measurements at each step.

The SBS-induced noise has been evaluated at the center of the Brillouin spectrum in dependence on the pump power for two different probe powers (−12 and −14 dBm). The result is shown in Figure 7. According to the theory, the P-I noise is zero in the center of the Brillouin spectrum. Thus, the SpBS beating noise is presented. For gain-and loss-based BOTDA, the noise is proportional to the probe power. Thus, due to the dependence of the probe and therefore the beating noise on the pump power (see Equations (5) and (11)), the SpBS induced noise for gain and loss-based BOTDA increases with pump power (see Figure 7). Additionally, as reported in the analysis in [27], the laser linewidth can introduce P-I noise even at the BFS, which leads to slight variations of the P-I noise with the pump power.

### 5.4. Spatial Resolution Dependency

**Figure 8:**
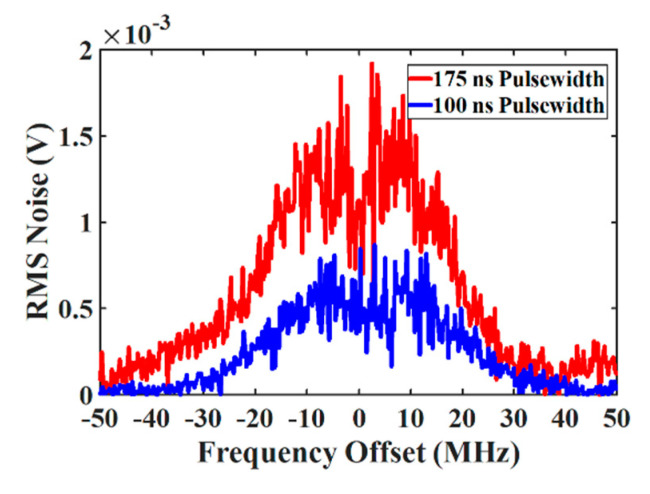
Experimental SBS-induced noise measurement in G-BOTDA for 10- and 17.5-m spatial resolutions.

To investigate the effect of the spatial resolution on the noise in gain-based BOTDA, the SBS-induced noise has been experimentally measured for a pump pulse width of 100 and 175 ns, which corresponds to 10- and 17.5-m spatial resolution, respectively, as shown in Figure 8. A lower spatial resolution leads to a longer Brillouin interaction and thus, a higher gain. This corresponds to increased beating and P-I noise. Therefore, the noise for a 17.5 m spatial resolution is higher than for a resolution of 10 m.

### 5.5. Noise Measurement in Frequency Domain

**Figure 9:**
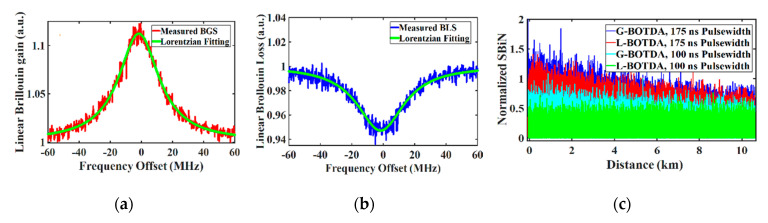
Measured Brillouin spectrum at a generic fiber position for 17.5 m spatial resolution: (**a**) gain case; (**b**) loss; (**c**) root mean square (RMS) noise measurement of the gain and loss case in the frequency domain along the fiber.

To investigate the noise in the frequency domain, the rms of the SBS-induced noise of the Brillouin gain/loss spectrum has been experimentally measured along the fiber. The rms noise level is calculated at each fiber position *z* according to:

(13) $$\sigma (z)=\sqrt[{}]{\frac{1}{k}\overset{k}{\underset{i=1}{\sum }}{\left(\left|\psi_{m}^{}(z)-\psi_{f}^{}(z)\right|\right)}^{2}}$$

where $\psi_{m}^{}$ is the measured Brillouin gain/loss spectrum, $\psi_{f}^{}$ is a Lorentzian fitted value and *k* is the number of frequency detuning steps. Thus, for the time domain noise measurements, for each frequency detuning step, the noise along the length of the fiber was measured (Equation (12)). For the frequency domain noise measurement instead, for each position *z* along the fiber, the noise of Brillouin gain or loss spectrum was measured (Equation (13)). Figure 9a,b shows the experimentally measured Brillouin gain/loss spectrum at a generic fiber position. The results reveal that the gain-based BOTDA has a higher noise level in the frequency domain along the fiber than the loss-based BOTDA at the same fiber section. The noise level is also higher for a longer pump pulse width as shown in Figure 9c.

The noise measurements in frequency and time domain are summarized in Table 2.

## 6. Conclusions

We have presented a complete theoretical model for the SBS-induced noise in BOTDA sensors, which mainly consists of probe-SpBS beating and P-I conversion noise. Simulation models for both gain and loss-based BOTDA have been developed. An experimental verification of the theoretical model has been carried out and shows good agreement with the simulation. Results reveal that loss-based BOTDA have a lower SBS-induced noise level than gain-based BOTDA. In gain-based BOTDA with 175 ns pump pulse width, the SBS-induced noise has its maximum at 10 MHz detuning from the BFS and the noise equals to 1.52 ×$\;10^{-3}$ V, while the noise reaches its minimum at the BFS and is 0.71 × $10^{-3}$ V. However, in loss-based BOTDA, the SBS-induced noise is about 1.01 ×$\;10^{-3}$ V at 10 MHz detuning and it is equal to 0.45 ×$\;10^{-3}$ V at the BFS (please see Table 2 for the summarized measurements results). The rms noise of the Brillouin gain/loss spectra in the frequency domain has been experimentally measured along the fiber as well. The results show a good agreement with the measurements in the time domain.

## Figures and Tables

**Table 1:** Experimental parameters.

Parameter	Value	Unit
Fiber length	10.6	km
Pump pulse width	175	ns
Pump power	20	dBm
Probe power	−14	dBm
Photodiode responsively	0.95	A/W
Photodiode bandwidth	1	GHz
Laser linewidth	2	MHz
Laser RIN	−135	dB/Hz

**Table 2:** Summary of the experimental noise measurements.

Parameter	Frequency Offset	Pump Power	Probe Power	Setup	Pulse Width	Result
CN	10 MHz	20	−14	Gain-based	175 ns	$3.8\times 10^{-3}$ V
CN	10 MHz	20	−14	Loss-based	175 ns	$3.8\times 10^{-3}$ V
SBiN	10 MHz	20	−14	Gain-based	175 ns	$1.5\times 10^{-3}$ V
SBiN	0 MHz	20	−14	Gain-based	175 ns	$0.7\times 10^{-3}$ V
SBiN	10 MHz	20	−14	Loss-based	175 ns	$1\times 10^{-3}$ V
SBiN	0 MHz	20	−14	Loss-based	175 ns	$0.4\times 10^{-3}$ V
SBiN	0 MHz	17	−14	Gain-based	175 ns	$\;0.4\times 10^{-3}$ V
SBiN	0 MHz	17	−14	Loss-based	175 ns	$0.1\times 10^{-3}$ V
SBiN	0 MHz	27	−14	Gain-based	175 ns	$\;2\times 10^{-2}$ V
SBiN	0 MHz	27	−14	Loss-based	175 ns	$2.9\times 10^{-3}$ V
*Noise ratio	Whole BGS	20	−14	Gain-based	175 ns	1
*Noise ratio	Whole BLS	20	−14	Loss-based	175 ns	0.8
*Noise ratio	Whole BGS	20	−14	Gain-based	100 ns	0.5
*Noise ratio	Whole BLS	20	−14	Loss-based	100 ns	0.2

Brillouin noise normalized to the noise of 175 ns gain-based BOTDA at 1 m fiber.

## Appendix A

Here, the analytical expression of the spontaneous Stokes-probe wave beating noise described by Equation (6) is derived. The same procedure can be followed to derive the analytical expression of the spontaneous anti Stokes-probe wave beating noise in loss-based BOTDA where the probe frequency upper-sideband and the anti-Stokes component are detected. From Equation (3), the total optical field reaching the PD in gain-based BOTDA will be:

(A1) $$E_{t}(t)=E_{s}^{+}e^{i\left[\left\{\omega_{p}-\left(\omega_{BFS}\pm \Delta \omega \right)\right\}t+\phi_{B}^{+}(t)\right]}+E_{spBS}^{S}e^{i\left[\left(\omega_{p}-\omega_{BFS})t+\phi_{SpBS}^{+}(t\right)\right]}$$

The current generated after the PD is given by:

(A2) $$I_{GBOTDA}(t)=ℜ\cdot {\left|E_{t}(t)\right|}^{2}=ℜ\cdot {\left|E_{s}^{+}e^{i\theta_{B}^{}}\right|}^{2}+ℜ\cdot {\left|E_{SpBS}^{S}e^{i\theta_{SpBS}^{}}\right|}^{2}+2ℜ\cdot \left|E_{s}^{+}e^{i\theta_{B}}\right|\left|E_{SpBS}^{S}e^{i\theta_{SpBS}^{}}\right|$$

where $\theta_{B}^{}$ and $\theta_{SpBS}^{}$ are the phase of the detected probe wave and the spontaneous Stokes component, respectively. Let $ℜ\cdot {\left|E_{s}^{+}e^{i\theta_{B}^{}}\right|}^{2}$ be the current of the amplified probe sideband, $ℜ\cdot {\left|E_{spBS}^{S}e^{i\theta_{SpBS}^{}}\right|}^{2}$ the spontaneous Brillouin component current and $2ℜ\cdot \left|E_{s}^{+}e^{i\theta_{B}}\right|\left|E_{spBS}^{S}e^{i\theta_{SpBS}^{}}\right|$ the probe-SpBS beating current, Equation (A2) yields [48]:

(A3) $$I_{GBOTDA}=I_{B}+I_{SpBS-SpBS}+I_{B-SpBS}$$

The SpBS-induced current noise originates from the fluctuations due to the beating between the probe and the SpBS and the beating between SpBS with itself. The variance of the current of $I_{GBOTDA}$ in Equation (A3) can be calculated as:

(A4) $$\sigma^{2}{}_{SpBS}=\langle I_{GBOTDA}^{2}\rangle -{\langle I_{GBOTDA}^{}\rangle }^{2}$$

(A5) $$\begin{array}{l} =\langle {\left(I_{B}+I_{B-SpBS}+I_{SpBS-SpBS}\right)}^{2}\rangle -{\langle \left(I_{B}+I_{B-SpBS}+I_{SpBS-SpBS}\right)\rangle }^{2}\\ =\underset{\sigma_{B-SpBS}^{2}}{\underset{︸}{\langle I_{B-SpBS}^{2}\rangle -{\langle I_{B-SpBS}^{}\rangle }^{2}}}+\underset{\sigma_{SpBs-SpBS}^{2}}{\underset{︸}{\langle I_{SpBS-SpBS}^{2}\rangle -{\langle I_{SpBS-SpBS}^{}\rangle }^{2}}}+\underset{\left(I_{B-SpBS},I_{SpBS}\right)}{\underset{︸}{2\left[\langle I_{B-SpBS}^{}I_{SpBS-SpBS}^{}\rangle -\langle I_{B-SpBS}^{}\rangle \langle I_{SpBS-SpBS}^{}\rangle \right]}} \end{array}$$

The first term of Equation (A5) represents the variance of the current due to the beating between the probe and the SpBS, the second term is the variance due to the beating between SpBS with itself and the third term is the correlation between the beating current and the SpBS. The second term can be neglected due to the low SpBS power, while the third term is set to zero since the probe and SpBS are uncorrelated, which is true for the random SpBS that is generated from random thermal noise in the material. Therefore, Equation (A5) becomes:

(A6) $$\sigma^{2}{}_{SpBS}=\sigma_{B-SpBS}^{2}=\langle I_{B-SpBS}^{2}\rangle -{\langle I_{B-SpBS}^{}\rangle }^{2}$$

To develop an analytical expression of the variance of the probe-SpBS beating current, from Equations (A2) and (A3), the beating current is given by:

(A7) $$I_{B-SpBS}=2ℜ\cdot \left|E_{s}^{+}e^{i\theta_{B}}\right|\left|E_{spBS}^{s}e^{i\theta_{SpBS}^{}}\right|=2ℜ\cdot \left|E_{s}^{+}\right|\left|E_{spBS}^{s}\right|\cdot \cos(\Delta \theta )$$

where $\Delta \theta =\theta_{SpBS}-\theta_{s}$ and it is a random variable between 0, 2π. Substituting Equation (A7) in Equation (A6), we obtain:

(A8) $$\sigma^{2}{}_{SpBS}=\langle {\left[2ℜ\cdot \left|E_{s}^{+}\right|\left|E_{spBS}^{}\right|\cos(\Delta \theta )\right]}^{2}\rangle -{\langle \left[2ℜ\cdot \left|E_{s}^{+}\right|\left|E_{spBS}^{}\right|\cos(\Delta \theta )\right]\rangle }^{2}$$

Since the $I_{B-SpBS}$ is a random variable, its average is equal to zero (second term of Equation (A8)). Provided that $\langle {\left|\cos(\Delta \theta )\right|}^{2}\rangle =\langle \frac{1+\cos(2\Delta \theta )}{2}\rangle =\frac{1}{2}$, therefore the $\sigma^{2}{}_{SpBS}$ is given by:

(A9) $$\sigma^{2}{}_{SpBS}=2{ℜ}^{2}\cdot \left[\langle {\left|E_{s}^{+}\right|}^{2}\rangle \langle {\left|E_{spBS}^{s}\right|}^{2}\rangle \right]$$

Since the single-mode fiber has two polarization modes, the electric field envelopes in x- and y-polarization components can be written as:

(A10) $$\begin{array}{l} E_{s}^{x}=E_{s}^{+}\cdot \cos(\varphi_{s})\\ E_{s}^{y}=E_{s}^{+}\cdot \sin(\varphi_{s})\\ E_{spBS}^{x}=E_{SpBS}^{s}\cdot \cos(\varphi_{SpBS})\\ E_{spBS}^{y}=E_{SpBS}^{s}\cdot \sin(\varphi_{SpBS}) \end{array}$$

where $E_{s}^{x}$, $E_{s}^{y}$ represent the x-, y-polarization components of the probe wave and *φ_s_* is the polarization rotation angle of the probe wave. $E_{SpBS}^{x}$, $E_{SpBS}^{y}$ stand for the x-, y-polarization components of spontaneous Stokes wave and $\varphi_{SpBS}$ is the polarization rotation angle of the spontaneous Stokes wave. Equation (A10) can be rewritten as:

(A11) $$\sigma^{2}{}_{SpBS}=2{ℜ}^{2}\cdot \left[\langle {\left|E_{s}^{x}+E_{s}^{y}\right|}^{2}\rangle \langle {\left|E_{SpBS}^{x}+E_{SpBS}^{y}\right|}^{2}\rangle \right]$$

Given that the beating between any two orthogonal components are equal to zero and after further simplifications, Equation (A11) becomes:

(A12) $$\sigma^{2}{}_{SpBS}=2{ℜ}^{2}\cdot \left[\langle {\left(E_{s}^{x}E_{SpBS}^{x}\right)}^{2}\rangle +\langle {\left(E_{s}^{y}E_{SpBS}^{y}\right)}^{2}\rangle \right]$$

Substituting Equation (A10) in (A11), yields:

(A13) $$\sigma^{2}{}_{SpBS}=2{ℜ}^{2}\cdot \left[\langle E_{s}^{2}\cos^{2}(\varphi_{s})E_{SpBS}^{2}\cos^{2}(\varphi_{SpBS})\rangle +\langle E_{s}^{2}\sin^{2}(\varphi_{s})E_{SpBS}^{2}\sin^{2}(\varphi_{SpBS})\rangle \right]$$

Since both the polarization angles $\varphi_{SpBS}$ and $\varphi_{s}$ are random variables between [0, π/2] and by using the definitions $P_{s}={\left|E_{s}^{+}\right|}^{2}$ as the Brillouin amplified detected probe power, $P_{SpBS}={\left|E_{SpBS}^{s}\right|}^{2}$ as the power of the spontaneous Stokes component, Equation (A13) becomes:

(A14) $$\sigma^{2}{}_{SpBS}={ℜ}^{2}P_{s}P_{SpBS}$$

In the same way, the anti-Stokes-probe beating noise in loss-BOTDA can be derived, which has the same analytical expression of Equation (A14). Therefore, Equation (A14) can be written as:

(A15) $$\sigma^{2}{}_{SpBS}={ℜ}^{2}P_{G/L}P_{SpBS}$$
